# Estrogen Receptor Beta (ERβ): A Ligand Activated Tumor Suppressor

**DOI:** 10.3389/fonc.2020.587386

**Published:** 2020-10-23

**Authors:** Rahul Mal, Alexa Magner, Joel David, Jharna Datta, Meghna Vallabhaneni, Mahmoud Kassem, Jasmine Manouchehri, Natalie Willingham, Daniel Stover, Jeffery Vandeusen, Sagar Sardesai, Nicole Williams, Robert Wesolowski, Maryam Lustberg, Ramesh K. Ganju, Bhuvaneswari Ramaswamy, Mathew A. Cherian

**Affiliations:** ^1^Comprehensive Cancer Center, The Ohio State University, Columbus, OH, United States; ^2^Stefanie Spielman Comprehensive Breast Cancer, The Ohio State University, Columbus, OH, United States

**Keywords:** ESR1, ESR2, ERα, ERβ, breast cancer

## Abstract

Estrogen receptor alpha (ERα) and estrogen receptor beta (ERβ) belong to a superfamily of nuclear receptors called steroid hormone receptors, which, upon binding ligand, dimerize and translocate to the nucleus where they activate or repress the transcription of a large number of genes, thus modulating critical physiologic processes. ERβ has multiple isoforms that show differing association with prognosis. Expression levels of the full length ERβ1 isoform are often lower in aggressive cancers as compared to normal tissue. High ERβ1 expression is associated with improved overall survival in women with breast cancer. The promise of ERβ activation, as a potential targeted therapy, is based on concurrent activation of multiple tumor suppressor pathways with few side effects compared to chemotherapy. Thus, ERβ is a nuclear receptor with broad-spectrum tumor suppressor activity, which could serve as a potential treatment target in a variety of human cancers including breast cancer. Further development of highly selective agonists that lack ERα agonist activity, will be necessary to fully harness the potential of ERβ.

## Introduction

In 2020, 9–10 million cancer deaths are projected to occur worldwide (WHO). Despite tremendous progress in unbiased, high throughput analysis of genomic alterations in cancer, the identification of key drivers that can be successfully targeted for therapy remains a challenge. The continued dissection of signaling pathways in breast cancer, using well-designed pre-clinical experiments is essential for the development of effective new therapies.

Estrogens play a key role in cell growth and differentiation of primary and secondary reproductive organs (1). The actions of estrogens, including those of 17β estradiol, which accounts for the majority of biological estrogenic activity in human plasma, are mediated by estrogen receptors. These include the transcriptionally active estrogen receptors, including ERα and ERβ, and membrane localized estrogen receptors, namely the G protein coupled estrogen receptor (GPR30) and palmitoylated forms of ERα and ERβ that localize to the plasma membrane. Estradiol mediates long-term effects on cellular function through the transcriptional effects of ERα and ERβ, while the membrane localized estrogen receptors mediate rapid signaling effects. Both ERα and ERβ belong to the superfamily of nuclear receptors called steroid hormone receptors, which upon binding ligand dimerize, dissociate from the molecular chaperone HSP90, bind to DNA, either directly or through interaction with other transcription factors, and activate or repress the transcription of a large number of genes, thus modulating critical physiologic processes (2–6).

## Natural ERβ Ligands

As with ERα, estrogenic compounds including estradiol, estrone, and estriol activate ERβ. Relative to ERα, ERβ binds estriol and ring B unsaturated estrogens with higher affinity, while the reverse is true of 17β-estradiol and estrone (7–10). On the other hand, the dihydrotestosterone metabolites 5-androstenediol and 3β androstanediol are relatively selective (3-fold) for ERβ over ERα (11). The structures of these natural ligands and their binding affinities to ERα and ERβ have been shown in Table 1. These testosterone derived ligands of ERβ may be unique in their ability to recruit the transcriptional repressor CtBP (C-terminal Binding Protein) to promoters that include activator protein 1 (AP-1) binding sites, thus suppressing AP-1 mediated transcriptional up-regulation in microglia and astrocytes and downregulating inflammatory responses in the CNS (12). It also follows that a reduction in ADIOL levels may promote an inflammatory response. Furthermore, these effects are induced by Androstanediol (ADIOL) but not by 17β-Estradiol, suggesting that certain downstream effects may be ligand specific. This suggests that, similar to the case with ERα, ligands are best referred to as modulators rather than pure agonists or antagonists given that each ligand may induce a different output from the receptor depending on which binding partners are recruited.

**Table 1:** Structure of major the estrogen receptor ligands and their binding affinity for ERα and Erβ.

**Compound**	**Structure**	**Binding affinity (Ki, nM)**	
		**ERα**	**ERβ**
Estradiol	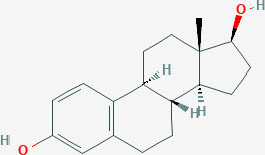	0.115 (0.04–0.24)	0.15 (0.10–2.08)
Estrone	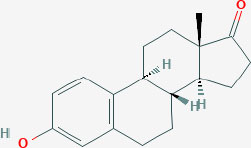	0.445 (0.3–1.01)	1.75 (0.35–9.24)
Estriol	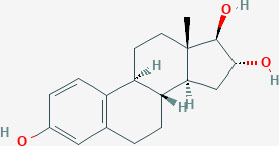	0.45 (0.35–1.4)	0.7 (0.63–0.7)
5-androsten-3β, 17β-diol	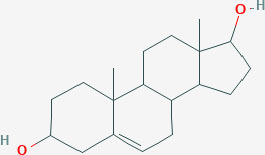	3.6	0.9
Genistein	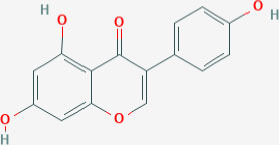	2.6–126	0.3–12.8
Daidzein	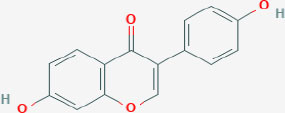	2	85.3
LY500307	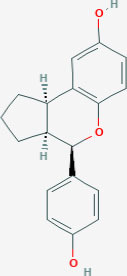	2.68	0.19

*All structures were obtained from PubChem: https://pubchem.ncbi.nlm.nih.gov/*.

## ERβ: Genetic Locus, Domain Structure, and Isoforms

**Figure 1:**
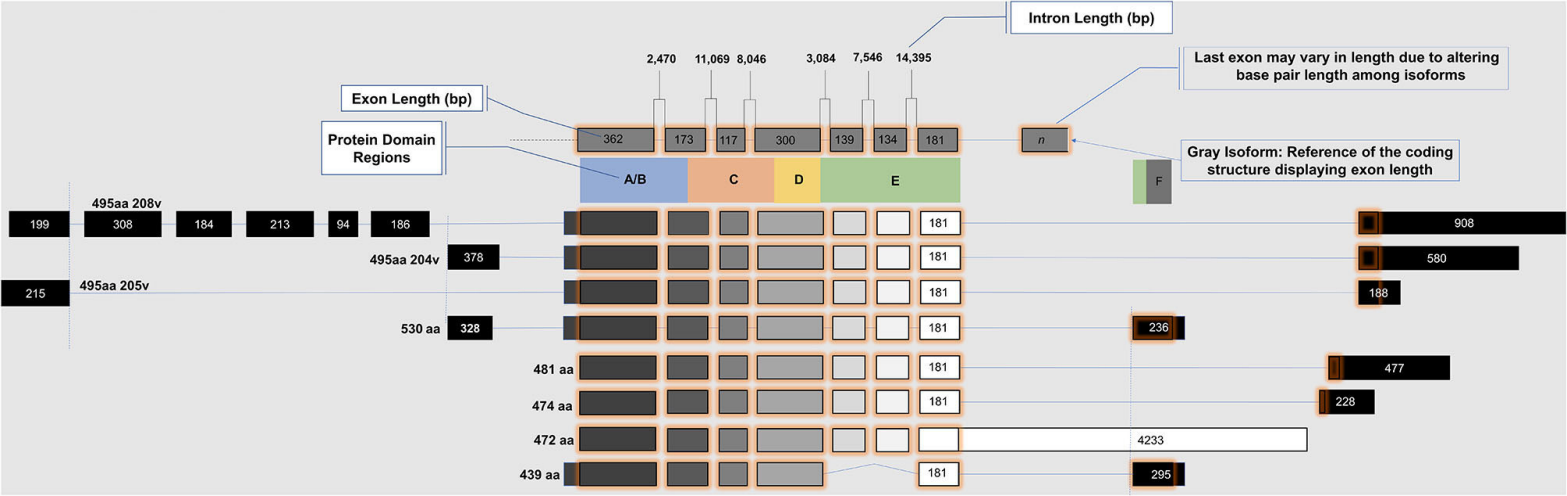
Exon map of *ESR2* isoforms.

**Figure 2:**
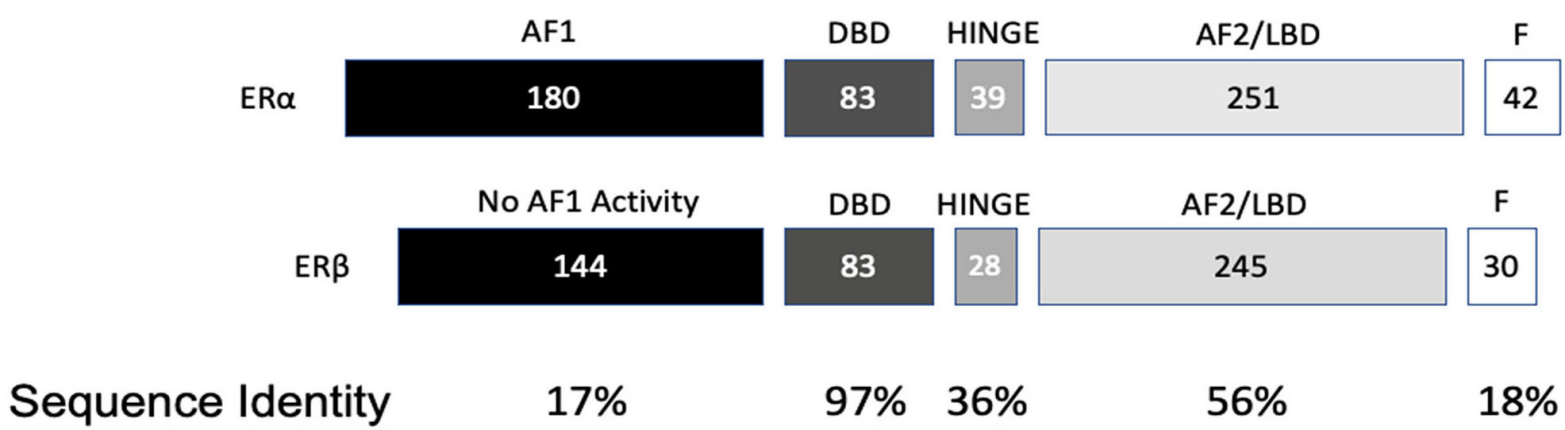
Protein domain similarity of ERα and ERβ.

*ESR2* (Estrogen Receptor 2), the gene that expresses ERβ, maps to 14q22-24, a distinct locus from that of *ESR1* (Estrogen Receptor 1), the gene that expresses ERα. *ESR2* spans 254 kb and contains eight encoding exons (13). The tertiary structure of ERα and ERβ includes an N terminal activation function-1 (AF1) or A/B domain that mediates weak ligand independent transcriptional activity; a C domain or DNA binding domain that binds to a cognate DNA binding element called an estrogen response element with high affinity, and also incorporates a weak dimerization capacity; a flexible D domain or hinge domain that includes a nuclear localization signal motif as well as a weak dimerization interface; the E domain or ligand binding domain that includes a ligand binding pocket and AF2 function as well as a dimerization interface and activates transcription in response to ligand binding; and a C-terminal F domain that negatively regulates ligand dependent dimerization (Figures 1, 2). Binding of ligand to the E/AF2/ligand-binding domain results in homo and heterodimerization with ERα and with other ERβ isoforms and dissociation from the chaperones HSP70 and HSP90 as well as cytoplasmic relocalization of HSP90 (2–6). Binding of anti-estrogens results in the formation of perinuclear clusters of the complex of estrogen receptor and ligand, while that of agonist results in persistent nuclear localization (6). Binding of ligands also results in association of a fraction of receptors with the small molecular chaperone HSP27, receptor acylation, augmented interaction with caveolin and membrane localization (14).

Domains of ERα and ERβ, protein domain function, and sequence identity are depicted in Figures 1, 2. There are multiple splice isoforms of ERβ (Figure 1). However, only the 530 amino acid full-length isoform possesses a complete ligand-binding pocket and is sensitive to activation by estradiol and other ligands. The different isoforms of ERβ have differing associations with prognosis. For example, ERβ1 tissue expression levels are considered to be lower in aggressive cancers (15–19). Interestingly, high ERβ1 expression is associated with a higher overall survival in women with breast cancer (20, 21). In contrast, ERβ2 over-expression, in certain contexts, is associated with poor outcomes (22).

## Post-Translational Modifications of ERβ Receptor

Post-translational modifications of estrogen receptors contribute to genomic and rapid non-genomic signaling, protein stability, dimerization, chromatin binding, and interaction with other regulators (23). The majority of these posttranslational modifications localize to the AF1 domain.

### Phosphorylation

Multiple amino acid residues of ERβ have been identified as sites of phosphorylation. Glycogen Synthase Kinase (GSK) induced Serine-6 phosphorylation primes ERβ K4 for increased sumoylation and decreased ubiquitination, these modifications being mutually exclusive and competitive. (24). Phosphorylation of tyrosine 36 is necessary for the tumor suppressor activity of the receptor in U87 glioblastoma cells, MCF7 breast cancer cells, and SKOV3 ovarian cancer cells. Interestingly, phosphorylation of Y36 of ERβ up-regulates its ubiquitination and turnover, suggesting that turnover may be necessary for transcriptional activity (25, 26). Similarly, phosphorylation of serine-60 and serine-75 residues increases the degradation of ERβ. Additionally, phosphorylation of serine-75 and serine-87 facilitates recruitment of the E3 ubiquitin ligase E6AP to the unliganded receptor, induces the transition of ERβ from inactive clusters to a more mobile state in the nuclear compartment, and increases its proteasomal degradation (27).

Interestingly, phosphorylation of ERβ at serines 75, 87, and 105, induced by ERK1/2 and p38 mitogen activated protein kinases (MAPK), augment the ligand dependent transcriptional activity of ERβ. Also of note, ERβ activation induces p38 MAPK activity that, in turn, induces ERβ expression and activity suggesting a positive feedback loop (28–30). A phosphomimetic mutant of S105 displayed enhanced transcriptional activity and cell migration and invasion.

A distinct phosphorylation site, Serine-106, is involved in recruitment of SRC-1 (also known as nuclear receptor coactivator-1), stimulation of estrogen receptor beta degradation, and activation of the transcriptional activity of ERβ. Phosphorylation of an additional serine residue, S124 also plays a role in the recruitment of SRC-1. Furthermore, phosphorylation of Tyrosine-488 also activates transcriptional activity of ERβ, and is homologous to the Y537 residue of ERα, a hotspot for activating mutations in breast cancer (31, 32).

A unique characteristic of ERβ is a phosphorylation site at serine 236 in the hinge region of the receptor that is mediated via Erb-B2/B3 and Akt activation, leading to the recruitment of the E3 ubiquitin ligase Mdm2 to ERβ, its polyubiquitination, and 26S proteasome mediated degradation. Phosphorylation of this negatively charged hinge region by Akt may also decrease the transcriptional activity of ERβ (33, 34).

### Ubiquitination and Sumoylation

The interaction of ERβ with the ubiquitin ligase E6AP enhances the transcriptional activity of the receptor, however, the specific target lysine for ubiquitination is yet to be described (27). Ligand dependent degradation by MDM2 E3 ligase has also been described but, similarly, the exact lysine residue has not yet been identified (27, 33). ERβ is also post-translationally modified at lysine 4 by small ubiquitin like modifiers. Glycogen Synthase Kinase (GSK)-induced serine-6 phosphorylation primes ERβ K4 for increased sumoylation and decreased ubiquitination, these modifications being mutually exclusive and competitive, causing increased and decreased protein stability, and decreased and increased transcriptional activity respectively (24, 27). ERβ sumoylation is also up-regulated downstream of Ras-MAPK signaling (24). The E3 ubiquitin ligase CHIP ubiquitinates ERβ, resulting in basal turnover and inhibition of CHIP with small molecules such as Diptoindonesin G (Dip G) causes stabilization of ERβ (35).

### Palmitoylation

Palmitoylation of ERβ at cysteine-399 by palmitoyl transferases is necessary for its plasma membrane localization (36). Experiments suggest that palmitoylation of ERβ may be obligatory for its membrane localization and binding with caveolin-1. Binding to caveolin-1 is necessary for the activation of p38 MAPK mediated pro-apoptotic effects of ERβ (36). Thus, palmitoylation may be a key post-translation modification for ERβ mediated pro-apoptotic effects. Interaction with the small heat shock protein HSP27 is also necessary for membrane localization (14).

### O-Linked N-Acetyl N-Acetyl-D-Glucosamine Glycosylation

O-Linked N-acetyl glucosamine glycosylation and O-linked phosphorylation of serine-16 of ERβ has been demonstrated to contribute to the stability of the protein: The wild-type estrogen receptor beta turns over rapidly with estrogen treatment (7–8 h half-life) as compared to the S16 mutant with an increased half-life (15–16 h). However, O-glycosylation at the same residue results in stabilization of the protein, suggesting that phosphorylation and O-glycosylation have opposite effects on protein stability (37). Another study showed that Serine-60 can also be modified through O-linked GlcNAcylation; this residue is located in the PEST region, which mediates rapid degradation of a number of different proteins, suggesting that this modification may regulate ERβ stability (38).

## Mechanism of Tumor Suppressor Function: Key Differences of ERα and ERβ

Given that ERβ and ERα are both expressed in many tissues, one key question is whether they act synergistically or antagonistically. The classic pathway of estrogen action includes ligand binding to the ER ligand-binding pocket causing a conformational change that induces dissociation of the ER-heat shock protein-90 (HSP90) chaperone complex receptor dimerization and binding to DNA (2). Ligand bound ER can also interact with other *trans*-acting factors, both co-transcriptional activators, and repressors, that modulate transcription. One difference between ERβ and ERα is the decreased transactivation potential of the ERβ AF1 domain and reduced cooperative interaction with its AF2 domain (39). Of note, unlike ERα, ERβ binds to the co-repressor nuclear corepressor (NcoR) in the unliganded state resulting in a lower basal level of transcriptional upregulation when expressed in the absence of ligand (40).

Binding of ER to estrogen response elements (ERE) within promoters and enhancers induces transcription. These *cis*-regulatory sites interact with the estrogen receptors in several different modes (41, 42). First, the receptor itself can bind to its cognate DNA binding element, the ERE, directly. However, the EREs exist in multiple conformations: consensus EREs (palindromic ERE repeats), non-consensus EREs, single binding site EREs, multiple binding site EREs, and composite ERE sites. In comparison to ERα, ERβ has reduced binding to non-consensus EREs, which represent the majority of estrogen responsive elements (e.g., C-*fos*, c-*jun*, pS2, cathepsin D, and choline acetyltransferase cis regulatory sequences). Estrogen receptors can also interact with DNA indirectly through tethering to other DNA-bound transcription factors like AP-1 (42, 43). In fact, genome-wide mapping of ERβ binding sites reveals extensive overlap with AP-1 binding sites (44). I a study by Hall and McDonnell, it was found that in transient transfection assays ERβ antagonizes the effect of ERα on certain E2 responsive E2-AP-1 composite promoters. This suggests that selective ERβ activation could decrease proliferation induced by ERα (19). Studies by Kim et al. (45) demonstrated the ligand and cell context dependent ERα/SP1 and ERβ/SP1 action using an estradiol responsive promoter containing three tandem GC rich Sp1 binding elements. Both ERα and ERβ proteins were found to interact with SP1 via their C-termini. ERα mediated transcriptional activation of the GC rich promoter element was observed in 3 out of 4 cell lines tested, but ERβ failed to activate transcription from the same promoter. Exchange of AF1 domains of ER subtypes gave chimeric ERα/β and ERβ/α proteins that resembled wild type ER in terms of physical associations with the SP1 protein. Transcriptional activation studies with chimeric ERα/β and ERβ/α showed that only ERα/β chimeras that included the ERα AF1 domain activated transcription from the GC rich promoter element. This suggests that only the AF1 domain of ERα is capable of productive interaction with SP1. In fact, a specific region of the AF1 domain, including amino acid residues 79–117 of ERα, was identified as being necessary for transcriptional activation from an SP1 binding element. In contrast, ERβ represses transcription of ERα by binding Sp1 and recruiting a corepressor complex containing NCoR to the ERα gene (*ESR1*) promoter (46).

The transactivation properties of ERα and ERβ were also examined with different ligands in the context of an estrogen response half element in tandem with a AP-1 binding element (47). Upon treatment with estradiol, ERα was found to upregulate transcription from this composite promoter. In contrast, ERβ was found to down-regulate transcription from the same promoter in the presence of estradiol, suggesting opposing effects for the two receptors at ERE-AP-1 composite response elements.

The cell division protein cyclin D1 (*CCND1*), one target of AP-1 and SP1 mediated transcription, is upregulated by ERα and induces estrogen-mediated proliferation (48). The *CCND1* promoter contains a cAMP response element and an AP-1 binding site, both of which play partially redundant roles in ERα mediated transcriptional up-regulation of cyclin D1. Surprisingly, activation of this promoter by ERβ was shown only to occur with antiestrogens. Estradiol, which up-regulates cyclin D1 transcription in cells that over-express ERα, inhibits its transcription in cells that over-express ERβ. Additionally, it was found that the presence of ERβ inhibits transcriptional up-regulation of cyclin D1 by ERα in the presence of estradiol, suggesting that ERβ may exert dominant negative effects on ERα mediated cyclin D1 transcription. Opposing actions and dominance of ERβ over ERα with respect to activation of cyclin D1 gene expression may explain why ERβ is a negative regulator of the proliferative effects of estrogen.

It is important to note that ERβ knock down in ER positive breast cancer cell lines induces an invasive phenotype, increases anchorage independent proliferation, and elevates EGFR signaling. This contrasts with the effects of ERα on EGFR signaling, which are collaborative in nature, and could explain how ERβ induction decreases anchorage independent proliferation and invasiveness (49, 50).

Human MutS homolog-2 (hMSH2) is a tumor suppressor mutated in hereditary non-polyposis colorectal cancer. Both ERα and ERβ interact with mismatch repair proteins, although the nature of the interactions diverge: ERα and hMSH2 interact in a ligand-dependent fashion, while ERβ and hMSH2 interact in a ligand-independent manner (51). In transient transfection assays, hMSH2 potentiated the transactivation function of ligand bound ERα but not that of ERβ. This suggests that hMSH2 may have a role as a coactivator of ERα dependent gene expression but not that of ERβ.

Another key difference is their interactions with steroid receptor co-activator 3 (SRC-3)/AIB-1, an oncogenic co-activator in both endocrine and non-endocrine cancers (52). SRC-3 expression was positively associated with ERα expression, while being inversely associated with that of ERβ (53). In the same study, HER2 positive primary tumor cell lines treated with tamoxifen induced SRC-3 recruitment to an estrogen response element and enhanced the interaction of SRC-3 with ERα but not ERβ. The selective recruitment of SRC-3 to cis-regulatory elements by ERα but not ERβ in the presence of tamoxifen could explain some of the previously studied differences between the two estrogen receptors in relation to cancer outcomes (53).

Estradiol increases the proliferation of and causes tumor formation of MCF-7 breast cancer cell line xenografts in an ERα dependent manner (54). In contrast, the over-expression of ERβ in MCF7 cells reduces proliferation *in vitro* and prevents tumor formation in mice in the presence of supplemental estradiol. Furthermore, ERβ was shown to repress c-myc and cyclin D1 expression, and to increase the expression of p21 and p27Kip1, leading to G2 cell cycle arrest in this model. In addition, ERβ regulates proliferation and migration through modulation of mitofusin 2 expression in MCF7 cells (54). Moreover, estrogen up-regulates the expression of LRP16 mRNA through the activation of ERα, but not ERβ, which promotes MCF-7 cell proliferation (55). Thus, ERβ and ERα have shown opposing effects on proliferation and the expression of various oncogenes and tumor suppressors in breast cancer cell lines in the presence of estradiol.

Malignant pleural mesotheliomas with high levels of AKT1 expression are associated with a worse prognosis, according to a study done by Pinton et al. (56). An inhibitory feedback loop of ERβ and AKT was described, suggesting an ERβ based therapeutic strategy for this malignancy. Interestingly, patients with high levels of AKT1 expression in tumors had a worse prognosis. These experiments demonstrated the role of the AKT1/SIRT1/FOXM1 axis in the repression of ERβ expression. Conversely, ERβ agonists increase the acetylation and inactivation of AKT1 in breast cancer (57). Other groups have demonstrated that the growth inhibitory effects of ERβ are repressed by ERK1/2 activation and PI3K-Akt activation, and rescued, in this context, by small molecule inhibitors of ERK and PI3K (58).

An additional opposing relationship between ERβ and PI3K-Akt activation occurs through the E3 ubiquitin ligase MDM2: heregulin-β, the ligand for HER2/HER3 dimers, activates Akt and triggers the recruitment of MDM2 to ERβ, leading to ERβ polyubiquitination and increased turnover (33). The ERβ transcriptional co-activator and histone acetyl transferase CBP stabilizes the interaction of ERβ with MDM2. Moreover, PI3K-Akt activation abrogates the potential for CBP to activate ERβ mediated transcription (33, 34). Conversely, ERβ increases active PTEN and reduces Akt phosphorylation when activated by estradiol (59). Thus, there are multiple lines of evidence suggesting an antagonistic relationship between ERβ and Akt signaling and the potential for synergy with PI3K-Akt inhibitors. These findings contrast with the effects of PI3K-Akt activation on signaling by ERα (60, 61). Interestingly, however, in certain contexts ERβ activation was shown to be associated with upregulation of serum and glucocorticoid dependent kinase (62) and PIM1(63) expression, both of which have some overlap with Akt in terms of function.

ERβ activation has been shown to modulate another target of the Akt pathway: the FOXO family of transcription factors. ERβ ligands 3β androstanediol, diaryl propiononitrile, and 8-vinylestra 1, 3, 5 triene-3,17-diol induce apoptosis via the p53 independent up-regulation of PUMA by FOXO3 (64). This transcriptional upregulation of FOXO3a by ERβ agonists is additional evidence for the potential for synergy with inhibitors of the PI3K-Akt pathway, activation of which results in phosphorylation of FOXOs by Akt leading to 14–3–3 binding, cytoplasmic retention, and degradation of FOXO transcription factors (65). Potential for therapeutic synergy between ERβ and FOXO transcription factors is further suggested by studies in prostate cancer models, which reveal transcriptional upregulation of FOXO1 by the un-liganded ERβ receptor (66, 67). These studies are unique in that they suggest that the un-liganded receptor is a tumor suppressor, and the estradiol bound receptor behaves like an oncogene in prostate cancer.

Other experiments suggest that non-genomic signaling by ERβ may be necessary for its tumor suppressor effects and this effect may involve interaction with and activation of p38 MAPK at the plasma membrane, effects that are distinct from those of ERα that predominantly activates ERK and PI3K-Akt through rapid non-genomic signaling (36, 68, 69). Thus, a key distinction between non-genomic effects of ERβ and ERα is the lack of activation of PI3K by ERβ (70).

Certain plant-derived flavonoids with xeno-estrogenic activity, such as naringenin, may synergize with estradiol in activating p38 MAPK, inducing caspase 3 activation, PARP cleavage, and apoptosis of human cancer cell lines that predominantly express ERβ (71). These same ligands selectively inhibit the non-genomic activation of ERK1/2 and Akt by ERα, without impeding its genomic action on an estrogen response element driven promoter (72–74). Similar effects with respect to rapid non-genomic activation of p38 MAPK have been described for the flavonoid quercetin, although this alkaloid induced p38 MAPK activation both through ERα and ERβ (22, 75). An artificial xenoestrogen, Bisphenol A, on the other hand, has the opposite effect, activating ERα dependent rapid membrane proximal signaling to ERK1/2 and Akt while inhibiting ERβ induced p38 MAPK activation, which may explain some of the endocrine disrupting effects that have been attributed to this compound (74, 76, 77). It is also important to note that ERβ activation induces p38 MAPK activity and p38 MAPK activity induces ERβ expression (36), and activity (29), suggesting a positive feedback loop.

Likewise, several other natural-product estrogen receptor ligands have estrogenic effects and may have chemo-preventive effects in breast cancer (78). For example, the isoflavone compounds genistein and daidzein that are present in soy-products have weak estrogen-like effects (Table 1) (79). Studies have shown that both these compounds at low concentrations increase the proliferation of ERα+ MCF7 breast cancer cells while at higher doses are inhibitory. In contrast, in ERα-ve cells (MDA-MB-231) this biphasic effect is not observed. Rather, both of these phytoestrogens demonstrate an anti-proliferative effect. This data indicates that both genistein and daidzein exert their proliferative effect (at low doses) through ERα while ERβ, which is expressed at low levels in both ER+ and ER– cells, opposes these actions and causes anti-proliferative, anti-invasive and anti-migratory effects as well as profound metabolic effects (80).

Studies with breast cancer cells have also demonstrated key differences in the interactions of estrogen receptors with TP53: ERα recruits SUV39H1/H2 to induce histone H3 lysine 9 trimethylation to silence TP53-activated transcription, an effect that is opposed by ERβ, which induces activating heterochromatin conformation by inducing histone H3 lysine 4 trimethylation and RNA polymerase II recruitment to ERα-repressed TP53-activated genes (81).

ERβ also binds to the aryl hydrocarbon nuclear translocator (ARNT) with increased affinity compared to ERα, suggesting that ARNT is a selective co-activator of ERβ (82). This association of ERβ with ARNT may lead to unique effects on hypoxia inducible factor-1α mediated upregulation of angiogenic factors via downregulation of aryl hydrocarbon nuclear translocator levels, leading to the inhibition of hypoxic signaling and angiogenesis (82–84). Loss of ERβ in prostate cancer induces transcriptional down-regulation of prolyl hydroxylase 2, resulting in decreased HIF1-α hydroxylation, and thereby decreased ubiquitination by the von Hippel-Lindau tumor suppressor and increased HIF1α protein expression (85).

An additional difference between ERβ and ERα lies in the capacity for ERβ to interact with HSP27 (86). Another unique protein-protein interaction of ERβ is that with MNK2 isoform b, which is of interest given the role of MNK kinases 1 and 2 in stimulating protein translation when concentration of eIF4A components such as eIF4E may be rate limiting (87). This interaction is unique to ERβ and for the b isoform of MNK2, but not ERα or MNK1. On the other hand, estradiol induced activation of AMPK has been shown to be mediated by selective interaction of the alpha catalytic subunit of AMPK with ERα but not ERβ (88). Any functional effects on protein translation or cell viability remain to be determined.

One gene that was shown to be selectively upregulated by ERβ and not by ERα, is TFGβ- inducible early gene-1/ Kruppel Like Factor 10 (KLF10) (89). Consistent with the low degree of sequence conservation in the AF1 domains of ERα and ERβ, this effect is mediated by the ERβ AF1 domain. The protein product of this gene induces apoptosis in pancreatic cancer cell lines (90). This decrease in cell viability may be due to induction of oxidative stress (91).

Another notable difference between ERα and ERβ are their effects on the unfolded protein response (UPR). The ERβ specific agonist ERB-041 has been shown to attenuate UPR associated IRE1α and XBP1 splicing induced by tunicamycin, a classic chemical activator of UPR, and, conversely, UPR downregulates ERβ expression (92). In addition, ERβ1 was shown to sensitize breast cancer cells to endoplasmic reticulum (ER) stress by attenuating XBP-1 splicing and inducing the degradation of IRE1α by upregulating its E3 ligase synoviolin 1 (93). In contrast, spliced and unspliced XBP-1 induce ligand independent ERα transcriptional activity (94). Additionally, XBP-1 is an ERα upregulated gene in ERα expressing cell lines (95). Key differences between ERα and ERβ are summarized in Table 2.

**Table 2:** Key differences between the action of ERα and ERβ on several genes or proteins.

**Effect on genes/proteins**	**ERα**	**ERβ**
ERE-AP-1	Upregulates transcription	Downregulates transcription
ER(α/β)/SP-1	Transcriptional activation	No transcriptional activation
CCND1 transcription	Positive regulator	Negative regulator
EGFR signaling	Increases signaling	Decreases signaling
hMSH2	Increased transactivation function	No effect
SRC-3/AIB-1	Enhanced interaction in presence of tamoxifen	No effect with tamoxifen
TP53	Silences TP53-activated gene transcription	Induces ERα-silenced-TP53-activated gene transcription
Serum and glucocorticoid dependent kinase	Upregulates SGK3	Upregulates SGK1
Unfolded protein response	Up-regulates XBP-1 expression	Induces degradation of IRE1 and downregulates XBP-1 splicing
Non-genomic effect	Activates PI3-Akt and ERK signaling—enhances tumorigenicity	Activates p38 MAPK signaling—enhances pro-apoptotic/tumor suppressor function

## High-Throughput Analysis of ERβ Action

Genome wide studies of ERβ induced transcriptional effects have shown clear differences from those of ERα. Using derivative sub-lines of the ERα+ breast cancer cell line T47D, stably transfected with a Tet-off ERβ expression construct, Williams et al. showed that of the 1434 transcripts whose expression was altered by ERα, ERβ expression negatively impacted ERα induced modulation of 998 (96). Surprisingly, they noted that expression of a DNA binding domain-deleted form of ERβ resulted in the same effect on ERα induced gene expression for many of these genes. Leucine-rich repeat-containing 15 and Apolipoprotein D mRNAs were markedly up regulated in estradiol treated ERβ expressing cells. ERβ markedly downregulated other genes such as the cytokine IL-20 and the anti-apoptotic gene Bcl2.

A similar study by Lim et al. (97) again in T47D cells, identified a number of genes whose upregulation by estradiol was inhibited upon ERβ overexpression including XBP1, PCNA, RAC1, CDK1, Cdc6, DNA replication helicase 2-like, and cell division cycle 28 protein kinase regulatory subunit 2. Studies by Chang et al. (98) in MCF7 cells showed that ERβ induces the expression of S100P while attenuating estradiol induced uregulation of FOXM1, CDC25A, E2F1, and survivin/BIRC5. Yet another study in MCF7 cells showed that both ERβ1 and ERβ2/ERβcx downregulated the expression of the proliferation related genes cathepsin D and IGFBP4 (99).

Secreto et al. (63) did similar experiments using Tet-inducible ERα and ERβ over-expression in the HS578T triple negative breast cancer cell line. In this study, treatment of doxycycline induced ERβ expressing cells with estradiol but not tamoxifen resulted in decreased proliferation and also modulation of expression of a number of genes. These included the downregulation of cyclin E2, connective tissue growth factor, Jagged1, and the upregulation of complement component 3, nuclear receptor interacting protein 1, CXCL14, and surprisingly, Pim1.

Another study in U2OS osteosarcoma cells showed a different sub-set of ERβ regulated genes, including upregulation of autotaxin and cystatin D (100). Other studies of ERβ induced gene expression in U2OS cells reveal quite marked differences in transcriptional effects of different ligands, suggesting that each different agonist may have distinct effects (101). These ligand dependent differences in ERβ induced transcription were further emphasized in a study that examined gene expression in ERα and ERβ overexpressing U2OS cells in response to estradiol, tamoxifen, and raloxifene (102). In this study, there was only a small overlap between ERα and ERβ regulated genes (17%). Additionally only 27% of genes were regulated by both tamoxifen and raloxifene in ERα and ERβ expressing U2OS cells, further emphasizing the potential for unique transcriptional effects of individual ligands. Among the genes that were markedly upregulated by ERβ was the IL-10 family member Mda-7/IL-24. This cytokine signals via the IL20 receptor to induce bystander cell cytotoxicity in cancer cell lines but not untransformed cells (103). The findings in regard to upregulation of Mda-7/IL20 by ERβ activation was confirmed in another study of U2OS cells that overexpress ERβ (104).

## Cell Non-Autonomous Effects of ERβ

In addition to its role in directly suppressing the viability of cancer cells, ERβ affects cancer cells *in vivo* through cell non -autonomous mechanisms. Zhao et al. reported the effects of a highly selective agonist, LY500307 in a TNBC xenograft model in mice. They observed suppression of lung metastasis in this model and demonstrated increased IL-1β secretion by cancer cells upon exposure to LY500307, which resulted in increased neutrophil recruitment into tumors and regression (105). These results suggest that ERβ activation may recruit immune cells into the tumor microenvironment. Notably, estrogen induced ERβ activation has been shown to cause NLRP3 inflammosome activation—a key upstream pathway for IL-1β processing and secretion—in models of endometrial cancer, although a pro-neoplastic role was suggested in this model (106).

Interestingly ERβ was shown to play a pro-inflammatory role in the estrogen driven non-malignant gynecologic disorder, endometriosis. ERβ is expressed at many fold high levels in endometriotic tissue as compared to normal endometrium (107). Over-expression of ERβ enhances the growth of endometriotic lesions in mice and interacts with components of the NLRP3 inflammosome such as NALP3, caspase-1, and caspase 9 and induces the processing of pro-IL-1β to its active form (108). In addition, several other inflammatory mediators such as macrophage inflammatory protein 1α, macrophage inflammatory protein 2, IL-16, monocyte chemoattractant protein 5, B lymphocyte chemoattractant, and triggering receptor expressed on myeloid cells (TREM1) were upregulated upon ERβ over-expression in endometrial cells. However, other cytokines were down-regulated such as TNF-α, monokine induced by interferon gamma, macrophage colony stimulating factor (M-CSF), interferon gamma inducible protein 10/CXCL10, and keratinocyte chemoattractant/CXCL1.

Conversely, several studies do suggest an anti-inflammatory role for ERβ in other contexts. For example, ERβ activation by selective agonists was shown to suppress TNFα induced gene expression by recruiting the co-activator SRC-2 forming a repressive complex (109). ERβ activation suppresses NF-κB activation and secretion of multiple cytokines in prostate cancer cell lines (110). In addition, an ERβ selective agonist, LY3201 inhibited NF-κB activation and neuroinflammation in murine models of demyelinating disorders, as did 5-androsten-3β, 17β-diol /ADIOL, another ERβ agonist (12, 111). Thus, the ramifications of ERβ activation on immune activation should be analyzed separately in each disease context, keeping the possibility of ligand specific effects in mind, to determine the net effects on immune activation.

## ERβ Knockout Mice

Krege et al. (112) developed and characterized the first ERβ KO mouse model in the late nineties. These mice develop normally and are morphologically and histologically indistinguishable from littermates. ERβ^−/−^ female mice are sub-fertile, with smaller litters than WT mice, which is corrected by ovulation inducers suggesting defects in ovulation. ERβ^−/−^ male mice reproduce normally but older mice develop prostatic and bladder hyperplasia. This phenotype contrasts with that of ERα KO mice, which are infertile and display a complete absence of breast development in females and reproductive tract, gonadal and behavioral deficits in males and females (113–117). Further investigation of the ERβ KO phenotype revealed the development of age related myeloproliferative disease resembling chronic myeloid leukemia with lymphoid blast crisis (118) and prostatic hyperplasia and prostatic intraepithelial neoplasia in the ventral prostate (119), consistent with a tumor suppressor role. Moreover, in mammary specific Cre- p53^F/F^ERβ^F/F^ mice, loss of ERβ accelerated the onset of mammary tumors, establishing ERβ as a tumor suppressor in the mammary epithelium (120). Additionally, deficits were noted in mammary epithelial organization and differentiation to secretory epithelium (121), colonic epithelial differentiation, and apoptosis (122), pulmonary development (123, 124), and neuronal migration in the developing cortex (118) of ERβ KO mice. ERβ KO female mice are partially protected from age-related trabecular bone loss (125), and have increased radial cortical bone growth (126), suggesting that maintenance of bone density is primarily a function of ERα.

## Regulation of Co-Transcriptional and Post-Transcriptional RNA Processing By ERβ

In addition to its effects on rapid non-genomic signaling and transcription, ERβ induces exon skipping of specific genes in response to estradiol (38). In breast cancer cells that were responsive to hormone therapy, there was a two-fold increase in splicing events in cells that expressed ERβ. ERβ also induced the promoter-switching of 61 genes and differential splicing of 28 genes and the transcription and splicing of transcriptional regulators such as *NCOR2*, which mediates gene repression by the tamoxifen-bound ERα (38).

## ERβ Expression and Role in Various Cancer Types

ERβ is unique in that it functions as a tumor suppressor in diverse biologic contexts. ERβ has been implicated in various cancer types, including breast, prostate, lung, glioblastoma, thyroid, and ovarian cancer (15–19).

Concerning breast cancer, ERβ expression by IHC is detectable in 20–30% of invasive breast cancers (127). In regard to association with patient outcomes in breast cancer, ERβ expression is an independent prognostic marker in ERα+-progesterone receptor positive breast cancer (128). Mann et al. (129) found that ERβ status is a predictor of survival in women with breast cancer when treated with adjuvant hormonal therapy. They also found that expression of ERβ in more than 10% of the cancer cells was associated with improved survival. This association has been confirmed in a meta-analysis showing improved disease-free survival in patients with tumors positive for ERβ1 and ERβ2 isoforms (130).

Although therapeutic efforts so far have focused more on triple negative breast cancer (TNBC), ERβ expression in tissue microarrays of breast cancer was significantly associated with expression of ERα and progesterone receptor and, when analyzed by molecular classification, higher in luminal A (72%) and luminal B (68%) breast cancers as compared to HER2+ and basal like sub-types (55 and 60%) (131). In this study, ERβ expression was inversely associated with HER2 and EGFR expression. These data suggests potential for therapeutic utility in ERα+ breast cancer. In ER+ve breast cancer ERα drives proliferation, while ERβ has anti-proliferative effects (11). Various mechanisms have been suggested. The attenuation of AP-1 and Sp1 mediated estradiol induced transcriptional activation is a potential, although not clearly established, mechanism for inhibition (132) and the AF1 domain of ERβ is necessary for this effect (133, 134). In regard to TNBC, Reese et al. suggested that ERβ induced the up-regulation of secreted proteins known as cystatins that down-regulate canonical TGFβ signaling, is critical for the suppression of the metastatic phenotype (135).

Some studies, however, suggest a pro-invasive effect of the weak and modestly selective ERβ ligand, di-aryl propionitrile, in triple negative SUM149 inflammatory breast cancer cells, an effect that MEK inhibition reversed (136). Another study suggested ERβ1 expression in TNBC is upregulated by insulin like growth factor 2 and was associated with a worse overall survival and that ERβ activation by diaryl propionitrile is associated with increased proliferation in TNBC and increased secretion of VEGF, amphiregulin, and WNT 10b/12 (137).

Hence, we still do not fully understand the mechanism of action of ERβ in breast cancer. Highly specific and selective agonists are required to fully characterize the role of ERβ in breast cancer, and, thus, determine its potential as a therapeutic strategy. The development of highly specific inhibitors is especially germane to the application of this strategy for breast cancers given the concurrent expression of ERα in the majority of breast cancers.

In the human male prostate, ERβ is expressed in stromal and luminal epithelial cells (138). Studies using ER-knockout mice have shown that mice lacking the ERβ gene develop prostate cancer at an increased rate with the addition of the pertinent hormones (139). ERβ may play a role in prostate differentiation and proliferation, and may modulate both the initial phases of prostate cancer as well as androgen-independent tumor growth (1).

Concerning the role of ERβ in lung cancer, the available data suggest both pro-tumorigenic as well as tumor suppressor roles depending on the experimental context. In healthy lung tissue, both pneumocytes and bronchial epithelial cells express ERβ. It is also required for the maintenance of extracellular matrix in the lung (140, 141). ERβ stimulates the proliferation of non-small cell lung carcinoma cells in certain experimental contexts (142, 143). Other reports suggest that cytoplasmic expression of ERβ is associated with therapy resistance to EGFR inhibitors (144). In addition, other studies have shown that nuclear ERβ is associated with a better prognosis, while cytoplasmic ERβ localization is associated with a poorer prognosis (145–147). When exclusively cytoplasmic, ERβ expression has been associated with increased growth of non-small cell lung cancer via extra nuclear non-genomic activation of cAMP, Akt, and MAPK signaling (148). Thus, in non-small cell lung cancer the effects of ERβ are complex and possibly differ from the classic tumor suppressor role it plays in other malignancies.

One study suggests that glioblastoma cells predominantly express the ERβ1 and ERβ5 isoforms, and the ERβ2 and ERβ4 isoforms to a lesser degree (149). ERβ knockout human GBM cell lines have increased invasive properties and re-introduction of ERβ1 reverses these effects. In contrast, the over-expression of ERβ5 actually increased anchorage-independent growth. *In vivo*, the expression of ERβ1 in U251-ERβ-KO GBM xenografts was associated with longer survival and was the only isoform with a tumor-suppressive effect.

Estrogen receptor beta may have therapeutic relevance in Hodgkin lymphoma. One article suggests that the ERβ agonist 2,3-bis (4-hydroxyphenyl)-propionitrile (DPN) could reduce Hodgkin lymphoma cell growth up to 60%. The mechanism of reduced cell growth was suggested to be via the overexpression of damage-regulated autophagy modulator 2 (DRAM2) and protein 1 light chain 3 (150).

Expression of estrogen receptor beta also seems to have an effect on patients with ovarian cancer. One study showed that patients with ovarian tumors that expressed cytoplasmic ERβ had prolonged survival compared to patients whose tumors did not express the receptor (151). Interestingly, another study concluded that nuclear expression of ERα or ERβ was not associated with clinical outcome (152). Pujol et al. further described expression of ERα and ERβ in ovarian cancer, normal ovarian tissue, metastatic tissue, and benign tissue (153). Normal ovarian tissue expressed ERβ at higher levels than in ovarian cancer cell lines. Seventy-eight percent of benign and borderline tumors expressed ERβ mRNA, whereas 29% expressed ERα. In another study of ovarian cancer, metastatic cancer tissues expressed only ERα mRNA and protein, suggesting that ERβ expression is down regulated at metastatic sites consistent with its role as a tumor suppressor (154). Lastly, Chan et al. (155) showed that ERβ1 expression is lower in high grade cancers and that higher cytoplasmic expression of ERα and ERβ1 is associated with better disease-free and overall survival. They also noted that higher nuclear ERβ5 and lower cytoplasmic ERβ5 was associated with clear cell histology and a worse prognosis.

An overwhelming amount of literature suggests that there is an inverse relationship between the expression of ERβ and the presence of colorectal polyps. The effects of ERβ in colorectal cancer may include reduced metastasis (via inhibiting PROX1), enhanced apoptosis (via p53), an anti-inflammatory response (via NF-kB), and reduced proliferation (by inhibiting c-myc) (156).

Zeng et al. (157) showed that the ERβ selective agonist DPN suppressed proliferation of papillary thyroid carcinoma (PTC) cell lines in contradistinction to the effects of the ERα specific agonist PPT, which increased proliferation. Similarly, Dong et al. (158) examined the effects of estradiol in the BCPAP PTC cell line and discovered that ERβ, in contrast to the effects of ERα, suppresses motility of these cells on exposure to estradiol. Reduced expression of ERβ1 in ERα negative PTC was associated with greater invasiveness and metastasis (159). Expression of the ERβ2 isoform in PTC was also associated with reduced lymph node metastasis in pre-menopausal patients with PTC (160). However, another study by Dong et al. observed that both nuclear, and combined nuclear and cytoplasmic staining for ERβ1, by immuno-histochemistry was lower in PTC compared to the lesions of a benign nodular thyroid goiter (BNTG) but that nuclear and combined nuclear and cytoplasmic staining for ERβ2 was actually higher in PTC than benign lesions (159, 161). Similarly, in the medullary thyroid carcinoma cell line TT, the expression of ERβ suppressed growth, again in contradistinction to the effects of ERα over-expression (162).

Renal cell carcinoma (RCC) cell lines express ERβ, which mediates estradiol-induced decreases in viability (163). Moreover, ERβ protein expression was lower in RCC than in normal kidney when examined by immuno-histochemistry. These findings suggest a tumor suppressor role for ERβ in RCC.

## Conclusions

The promise of ERβ activation lies in the possibility of a targeted therapy that concurrently activates multiple tumor suppressor pathways while causing relatively few side effects. Thus, ERβ is a nuclear receptor with broad-spectrum tumor suppressor activity that could serve as a potential treatment target in a variety of human cancers. Further refinement of selective ERβ modulators with the development of highly selective ERβ agonists that lack ERα agonist activity will be necessary to fully harness the potential of this promising therapeutic avenue. Gene expression signature and non-genomic signaling pathway activation should be defined for each compound given the potential for unique ligand dependent effects on the recruitment of binding partners (101). In addition, further investigation into the mechanism of action of ERβ agonist is required for the design of potential synergistic combinations with other therapeutic drug classes for breast cancer.

## Author Contributions

RM, AM, JDav, JDat, MV, MK, JM, NWillin, and MC conducted a review of the literature and prepared the body of the manuscript. DS, JV, SS, NWillia, RW, ML, RG, and BR critically reviewed the publication. MC drafted the manuscript. All the authors endorsed the final form of the manuscript.

## Conflict of Interest

The authors declare that the research was conducted in the absence of any commercial or financial relationships that could be construed as a potential conflict of interest.

## Abbreviations

ADIOL: Androstanediol
AF1: Activation Function-1
CCND1: cell division protein cyclin D1
CtBP: C-terminal Binding Protein
EGFR: epidermal growth factor receptor
ERβ: Estrogen Receptor β
ERα: Estrogen Receptor α
EREs: estrogen response elements
E6AP: E6 Associated Protein
MAPK: Mitogen Activated Protein Kinases
GSK: Glycogen Synthase Kinase
HER2: Human Epidermal Growth Factor 2
hMSH2: Human MutS homologue-2
HSP90: heat shock protein-90
KO: knockout
PARP: poly (ADP-ribose) polymerase-1
SRC-3: steroid receptor co-activator 3.
